# Genotype-driven recruitment: a strategy whose time has come?

**DOI:** 10.1186/1755-8794-6-19

**Published:** 2013-05-23

**Authors:** Isabelle Budin-Ljøsne, Kaitlin J Soye, Anne Marie Tassé, Bartha Maria Knoppers, Jennifer R Harris

**Affiliations:** 1 Division of Epidemiology, Department of Genes and Environment, Norwegian Institute of Public Health, P.O. Box 4404, Nydalen, Oslo NO-0403, Norway; 2 Centre of Genomics and Policy, McGill University, 740 Doctor Penfield Avenue, suite 5203, Montreal, Quebec H3A 1A4, Canada

**Keywords:** Genotype-Driven Recruitment, Feedback of results, Genetic testing, Biobanks

## Abstract

**Background:**

Genotype-Driven Recruitment (GDR) is a research design that recruits research participants based on genotype rather than based on the presence or absence of a particular condition or clinical outcome. Analyses of the ethical issues of GDR studies, and the recommendations derived from these analyses, are based on GDR research designs that make use of genetic information already collected in previous studies. However, as genotyping becomes more affordable, it is expected that genotypic information will become a common part of the information stored in biobanks and held in health care records. Furthermore, individuals will increasingly gain knowledge of their own genotypes through Direct-to-Consumer services. One can therefore foresee that individuals will be invited to participate not only in follow-up GDR studies but also in original GDR studies because genetic information about them is available. These individuals may or may have not participated in research before and may or may not be aware that their genetic information is available for research.

**Discussion:**

From a conceptual point of view, we investigate whether the current ethics-related recommendations for the conduct of GDR suffice for a broader array of circumstances under which genetic information can be available. Our analysis reveals that the existing recommendations do not suffice for a broader use of GDR.

**Summary:**

Our findings refocus attention on ethical issues which are neither new nor specific to GDR but which place greater demand on coordinated solutions. These challenges and approaches for addressing them are discussed.

## Background

Biobanks and genomic studies are accumulating large numbers of samples and generating databases that create novel research opportunities within the biomedical and behavioural sciences. An increasingly important design used in genetic research is called Genotype-Driven Recruitment (GDR) [1,2]. In GDR, research participants are not recruited based on the presence or absence of a particular condition (e.g. diabetes or high blood pressure) but are rather recruited based on their genotype characteristics such as the presence of a particular gene variant (e.g. a specific polymorphism or a gene deletion) [1,2]. GDR is a particularly useful design for studying the role of specific genotypes on health-related outcomes including differences in disease susceptibility, differential responses to interventions and treatments or normal variation in developmental outcomes. GDR studies have great potential to increase the utility of genomic data generated in diverse settings and thereby can accelerate the translational process towards benefiting human health [1-3].

The ethical implications of GDR have been recently discussed in the literature and recommendations have been made to address these issues [1-18]. However, these recommendations were derived from analyses of GDR designs that recruit via re-contact of individuals for whom genetic data already exists because they have participated in a study that collected and analyzed DNA. In this paper, we examine from a conceptual point of view, whether the ethics-related recommendations which have been proposed under circumstances of re-contact for GDR research sufficiently cover the ethical concerns that can arise as opportunities to use GDR increase with the growing availability of genotypic data. In particular, we focus on the ethical and societal implications of recruiting individuals in original GDR studies because genetic information about them is known or available through, for example, health care medical records or health records from Direct-to-Consumer (DTC) genetic testing services. These individuals may or may have not participated in research before and may or may not be aware that their genetic information is available for research.

### Potential future uses of GDR

Current studies employing GDR are primarily follow-up studies making secondary use of genetic information collected in previous studies [1-3,7,9,14,17]. As genotyping becomes more affordable, the use of GDR studies will most likely expand for two main reasons. First, individuals may be recruited into GDR studies because their genetic information is made available through the health care system. Several projects are currently planning to sequence entire populations or large groups of individuals and store their genetic information in healthcare databases. These projects are designed with the objective to 1) offer better disease diagnosis, therapy and clinical outcome for patients through integrated analysis of clinical, biological, environmental and genetic data [19] and 2) produce datasets that may be made available to national and international researchers through the approval of data access applications. For example, the FarGen project [20] plans to sequence the whole population of the Faroe Islands (approximately 50,000 individuals) within the coming years. The population of the Faroe Islands is particularly interesting for researchers because it is a small homogenous population with a high incidence of rare hereditary diseases [21]. It is therefore expected that the genetic information produced through the FarGen project will be an attractive resource to conduct GDR studies. Another example is the British National Health Service’s (NHS) plan to build a massive database in which every British citizen’s DNA records will be stored and made accessible to researchers from public and private institutions [22,23]. Both the Faroese and the British project plan that the genetic data produced will be stored in patient records and linkable to other health registries [20,23]. These types of collections are invaluable for conducting GDR studies that will play an important role in advancing the goals of precision medicine [19]. Although it is still unclear which requirements and limitations will apply for access to the data produced through these projects (e.g. access to coded or encrypted datasets), one can foresee that GDR will be in high demand and procedures to conduct GDR will need to be established.

A second reason why the potential to recruit individuals through GDR may increase is because an individual’s genetic information may be made available through DTC genetic testing companies. The last years have seen the emergence of a myriad of private companies which offer genetic screening services to consumers at an affordable price [24]. The privacy policies of these companies often do not guarantee that genetic data will not be shared with third parties and sometimes even state that genetic data may be shared for research purposes or sold in the event of company dissolution, merger or acquisition [24,25]. For instance, the DTC genetic testing company 23andMe is now seeking collaborations with academic researchers to maximise the scientific yield from their database [26]. The company highlights that research on rare genotype-phenotype combinations is one of the proposed areas of collaboration [26]. It is also foreseen that future business plans of DTC companies will give greater priority to selling DTC customer data to commercial entities to increase their return on investment [27,28]. Under such conditions, it is likely that the genetic data of the customers of DTC companies could be made accessible to researchers who want to conduct GDR studies.

If, as we believe, opportunities for GDR-based studies expand with the growing generation of genotypic data it is critical to investigate how this impacts the ethical landscape of GDR.

## Methods

A literature search was conducted using Pubmed and Google Scholar to identify studies that describe a) ethical issues in GDR-based research and, b) current recommendations to address those issues. Only articles which specifically discussed or mentioned the ethical aspects of GDR were selected from the search results [1-18]. An overview of the ethical issues identified and the recommendations made to address each of these issues was then compiled.

Next, a conceptual analysis was conducted that examined whether each of the ethical issues identified was sufficiently handled under the current set of recommendations for the cases where individuals were not *re*-*contacted* for a follow-up GDR study but contacted to participate in an original GDR study because their genetic information is available in public or private health care records. The information generated from this analysis is described herein and serves as the basis upon which we conclude whether the current recommendations suffice under the broader utilization of GDR.

## Results

Results of our literature review reveal that the main ethical issues associated with GDR are related to the re-contact of research participants and the disclosure to them of their genetic information during the recruitment phase [1-4,6,7,9,14,17]. Table 1 provides an overview of our analysis results including the ethical concerns identified, recommendations forwarded to address these, whether the current recommendations adequately address the ethical concerns raised under a broader utilization of GDR and the reasoning behind these determinations.

**Table 1:** Overview of ethical concerns in GDR and assessment whether current recommendations suffice for a broader use of GDR

**Ethical concerns in GDR**	**Published recommendations**	**Do the recommendations suffice under a broader use of GDR?**
Risk of violating the individual’s privacy and right not to know if genetic information is implicitly unveiled during re-contact [1,3,4]	Inform about potential re-contact and future disclosure in the informed consent of original study [2,6,7,12]	No: informed consent process may not have taken place before genotyping
	Offer the individual the possibility to choose whether she wants to be involved in future GDR studies [2-4,6,7]	No: choice to participate in future GDR studies may not have been given
	Design re-contact process in consultation with the Institutional Review Board (IRB) of the original study [6,7]	No: contact with/identification of the IRB of the original study may not be possible
Risk that the individual enrolled does not understand why she is eligible for the GDR study [9,14]	Take into consideration the context into which the individual is invited to participate in GDR (e.g. previous participation in research, previous research focusing on similar condition) [6,7,14]	No: the research background of the individual may be unknown to the researcher inviting to join a GDR study
Risk of creating unnecessary anxiety and distress for the individual re-contacted [1]	Contact by known and/or trusted health professional [3,4,7]	No: the individual may receive an invitation to participate in an original GDR study from unknown researchers
Risk of deceiving the individual if he is not informed about the reasons for enrolment [1]	Return genetic results to the individual more systematically than in other research designs [1,3,6,7,9,15,17]	No: the individual may not have been prepared to receive genetic results
Risk of group/individual discrimination and/or stigmatisation if re-identification is possible through the analysis of the study’s pedigree description [1]	Add randomly selected sub-groups in case-only studies [1]	Yes: randomly selected sub-groups could also be added in original GDR case-only studies
Risk of provoking emotional distress for the individual if the results disclosed are uncertified, have low clinical utility [1,3,5] or if they indicate the presence of a rare variant of significance for the health of the individual [1,4,9]	Return only results which are clear, concise, and accurate and explain the meaning and utility of the results [1,7,9,15,17,18]	No: individuals may not have been prepared to receive genetic results
	Use consistent disclosure criteria as agreed beforehand with individuals [8,15]	No: process to reach agreement beforehand on which results to return may not have taken place
	Design the return of results process in consultation with the IRB of the original study [7]	No: contact with the IRB of the original study may not be possible
	Take into consideration the context of the research, the relationship between research and research participant, and the degree of vulnerability and dependence of the research participant [5,7,9]	No: the personal/research background of the individual may be unknown to the researcher inviting to join a GDR study
Risk of making the individual re-contacted the carrier of bad news for non-contacted family members [1,4,9]	Include recruitment of family members as part of the research protocol [4]	No: individuals may not have had the opportunity to consent to the invitation of their relatives into the GDR study
Risk of harassing the individual if multiple re-contact for inclusion in GDR studies takes place [1]	Make use of independent governance bodies (e.g. centralised ethics committee or data access committee) which can determine which individuals to re-contact [2,10]	No: governance bodies may not be co-ordinated across publicly funded and privately funded projects
	Inform the individual about the role of governance bodies in the consent of the original study [10]	No: informed consent process may not have taken place before genotyping
	Make use of flexible and less intrusive electronic information exchange systems for communication with research participants [2]	No: communication systems may not be established between the researchers inviting to participate in a GDR study and the individuals receiving the invitation

### Ethical concerns in GDR

GDR based on the secondary use of previously collected genotype data requires that the research participants be re-contacted for the purpose of recruitment into the study. Because genotypic information is the basis for the recruitment, GDR may implicitly result in researchers providing participants with some information about their genotype as well as other information related to their medical or health status [1]. However, providing such information during recruitment might violate the individual’s privacy and right not to know about their own genetic information [1-4]. It may also create anxiety and distress for the individual who might think that something is wrong about her health since she has been re-contacted [1,2]. By contrast, not providing such information at recruitment may be perceived by the individual as a form of deception because she would not know why she has been enrolled in the study [1].

Issues surrounding the divulgence of genetic results are at the crux of the ethical considerations in GDR. Today, the return of research results from genetic studies to research participants is a highly debated topic among scientists, ethicists and policy makers [29-31]. Current guidelines typically sanction the return of genetic results if they are actionable or clinically useful for the research participants [18,32-34]. However, to harness the full scientific potential of GDR designs, it is quite likely that the genetic selection criteria for recruiting participants will not meet the requirements of being actionable or clinically useful. Not providing genetic results may mask the reasons why participants have been enrolled in the study [1,7], but disclosing genetic results requires certain types of guidance for the participant. This process is compounded when results are experimental, partly unreliable or uncertified and may have serious consequences for the research participants who may make further health-related decisions based on shallow scientific ground [1,3,5]. However, even when the results are verified in clinical labs, the finding of a rare variant could also have dramatic consequences for the individuals concerned because they may affect their health and make them the carriers of bad news for their family members [1,4,9].

Re-contact for recruitment into a GDR study may also put the individuals re-contacted and their relatives in a difficult situation, especially if the study design (e.g. case only study) allows outsiders to determine which genetic variant is under study [1,2]. Finally, there is a risk that individuals with rare genetic variants may begin to feel overwhelmed if they receive numerous invitations to join GDR studies [1,2]. This could, in the long term, discourage individuals from participating in research.

### Published recommendations

A number of recommendations have been made to address the issues surrounding the return of genetic results described above and primarily focus on optimising the informed consent process and developing mechanisms for feedback of results to research participants. This is illustrated by recommendations to 1) include information about potential re-contact and future disclosure in the original consent [2,6,7,12]; 2) offer participants the opportunity to choose whether or not they want to be re-contacted for future participation in GDR studies [2-4,6,7]; and 3) ensure that re-contact occurs by a known and trusted health professional [3,4,7].

In addition, it is recommended that independent governance bodies determine, in close co-operation with the Institutional Review Boards (IRB) of the original studies, which individuals to re-contact and how to re-contact them [2,6,7,10]. Other recommendations include 1) choosing a study design which prevents the involuntary disclosure of information about the genetic profile of the research participants to the outside world, e.g. by adding randomly selected sub-groups in case-only studies [1]; 2) using less stringent parameters for the return of results to research participants than currently recommended in other genetic research [1,3,6,7,9,15,17] and 3) return of results according to specific criteria agreed upon in co-operation with the research participants [8,15].

Finally, a general recommendation is that researchers who plan to conduct GDR studies should take into consideration the context of the research (e.g. was the original study conducted by different researchers than the follow-up GDR study?) and the relationship between the researchers and the individuals they plan to invite [5,7,9].

### Do the recommendations suffice under a broader use of GDR?

Results from our conceptual analysis reveal that out of 15 recommendations proposed in the current literature only one may suffice in the context of a broader use of GDR. The recommendation to add randomly selected sub-groups in case-only studies to avoid risks of stigmatisation when individuals are invited to GDR studies may also be applicable when GDR studies are original studies and not follow-up studies. For each of the remaining fourteen recommendations, we found one or several reasons why the current recommendations would not suffice in a context of broader use of GDR designs. These reasons are reported in Table 1 and can be summarised as follows.

### The individuals invited to enroll in an original GDR study may not be prepared to receive such an invitation

Current recommendations presuppose that, at some point in time, an informed consent process has taken place between researchers and participants; a process during which information about potential future enrolment in GDR studies has been provided and the opportunity for participants to indicate whether they want to be involved in future GDR has been given. Recommendations also suggest that re-contact should be undertaken by a known professional such as the principal investigator of the study to which the participant was originally recruited. However, this type of individual informed consent process may not systematically take place in conjunction with the generation of genotypic information. An example would be genotyping conducted through standard health care where informed consent may not have been required. Individuals who are not aware that genetic information exists about them will probably be totally unprepared if contacted for participation in a GDR study [14]. Even individuals who have previous knowledge about their genotype from DTC genetic testing services may not be aware that their genetic information potentially could have been sold to or shared with other private companies or researchers [25]. Thus, unexpected contact may come as a surprise and provoke anger if the individuals contacted do not want to know about own genetic information and do not want their relatives to know [1,3,4,9].

### The individuals invited to enroll in an original GDR study may not be prepared to receive genetic research results

When individuals who have participated in previous research are re-contacted for participation in a GDR study, they already have some knowledge about what a research study entails and what participating in research means. They have taken the initiative to participate in research and may have received some basic information related to the genetic research aims by the investigators of the original study. Things may be different for individuals who are not *re*-*contacted* but contacted for the first time; individuals who have never participated in research before and may not know anything about genetic research. There is currently no empirical evidence on the effects of receiving genetic results without having been prepared to it. One could argue that the disclosure of genetic research results to research participants normally does not trigger such distress as shown in recent studies [35,36]. However, the recipients of genetic results who participated in those studies already had a relationship with the researchers or were aware of the presence of a genetic variant in their family through a relative’s participation in research or illness. If individuals are recruited into original GDR studies through e.g. their health care system or directly by a private company, such a relationship and awareness may not exist.

Current recommendations also encourage applying a lower threshold than usual for feedback of results from GDR [1,3,6,7,9,15,17]. A sound feedback process that respects the perspectives and wishes of participants typically requires taking into consideration the context of the research and the relationship between the researcher and the research participant [8,15]. However, if as we postulate, GDR designs were used more broadly, it would not always be possible for researchers to make such an assessment because they may not know anything about the individual’s previous involvement in research.

### The establishment of independent governance bodies to coordinate all GDR research projects may be too challenging

The current recommendations encourage the establishment of independent governance bodies, e.g. centralised ethics or data access committees, by the projects generating genetic information to manage all data access requests, protect the privacy and confidentiality of datasets and determine which individuals can be contacted and by whom. The objective of establishing these committees is to avoid harassing individuals by inviting them to multiple studies as could be the case, for example, for individuals carrying a rare genetic variant, and ensure that ethical standards are respected [2,7,10]. Creation of such committees raises several sets of questions. First, questions related to the legal authority of these committees may emerge. For instance, under which legal framework, national or international, would these committees be established and operate? Could these committees have authority both over publicly financed research projects and private companies? As an example, DTC genetic testing companies currently do not operate under the same legal framework as publicly funded research projects [37] and, although attempts to regulate their activities have been made, they still benefit from a rather loose legal framework [38]. Unless international regulation requires that DTC genetic testing companies follow the same rules (and report to the same centralised committees) as publicly funded research projects, it is quite unlikely that they will do so.

Second, questions related to the scientific authority of these committees may emerge. How could these committees for instance make decisions regarding data access requests from numerous public and private actors? To do so would probably require that the committees have extensive knowledge about all research projects previously undergone, which individuals have participated in which research projects and which have not, which have consented to re-contact and which have not, which have already been invited in previous GDR studies, and which have not etc. Such a high level of co-ordination could maybe be possible between publicly-funded institutions within a specific research collaboration or geographic area [10] but seem quite unrealistic to expect when both publicly funded research projects and a myriad of private companies are involved.

## Discussion

We investigated whether current recommendations to address ethical issues in GDR suffice when GDR studies are original studies recruiting individuals from whom genetic information is available through, for example, health care medical records or health records from DTC genetic testing services. These individuals may or may not have participated in a research study and may or may not be aware that their genetic information is available for research. The current recommendations are useful when planning studies that will produce genotypic information of interest for future GDR. However, they were not developed to address potential issues that may arise if original GDR studies become more common due to the availability of individual genotypic information generated from a wider and more diverse array of sources.

From a conceptual point of view, we foresee that a broader use of GDR will call attention to a number of issues that are neither new nor specific to GDR per se but for which coordinated solutions will be strongly needed. Examples include:

1) How to deal with the dissemination of genetic information of uncertain value and utility towards groups of individuals that are unaware that such information exists about them and are insufficiently educated to understand the meaning of it;

2) How to widely disseminate information about genetic variants to individuals through the conduct of GDR studies without provoking the unnecessary medicalization of otherwise healthy individuals [39];

3) How to respectfully disseminate information about genetic variation to individuals about their own genotypes but which has implications at the familial level [4,17];

4) How to avoid provoking more genetic determinism and increasing potential risks of stigmatisation and discrimination of individuals when genotypes become a common selection criteria for invitation in research studies [40]; and,

5) How to avoid invitation-fatigue of individuals whose genotypes are highly sought after for biomedical research.

## Summary

There are growing opportunities for important research using GDR designs and recent studies show that research participants have a rather positive attitude towards GDR designs and understand their scientific value [3,9,15,17]. The recommendations that have been published to address ethical concerns in GDR are useful but should be complemented by other strategies to address new challenges for GDR designs that originate outside of a study-specific research setting. These strategies cannot be addressed by the researchers conducting GDR studies alone but need to be addressed at a broader level by health authorities, the scientific community from both public and private sectors, and policy makers. Such strategies may be bolstered by the development of tools to promote education, dissemination and public engagement. Examples include tools to: 1) widely inform the public about genetic research activities and the production of genetic information through health care systems and private companies; 2) provide all groups in society with basic education in genetics; 3) widely inform about the right to opt-out from research and the right not to know about one’s own genetic information; 4) protect and secure these rights in a co-ordinated manner across privately and publicly funded research projects, and 5) facilitate an increased involvement of the public in the design of research projects.

## Abbreviations

GDR: Genotype-Driven Recruitment; IRB: Institutional Review Board.

## Competing interests

The authors declare that they have no competing interests.

## Authors’ contributions

AMT, JRH, IBL and BMK conceived of the study. IBL and KJS drafted and critically revised the manuscript. AMT, JRH and BMK participated in its design, and critically revised the manuscript. All authors read and approved the final manuscript.

## Pre-publication history

The pre-publication history for this paper can be accessed here:

http://www.biomedcentral.com/1755-8794/6/19/prepub
